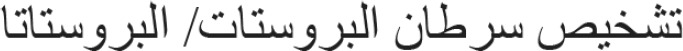# Quality and reliability of YouTube videos in Arabic as a source of patient information on prostate cancer

**DOI:** 10.3332/ecancer.2023.1573

**Published:** 2023-07-13

**Authors:** Laith Baqain, Deborah Mukherji, Humaid O Al-Shamsi, Ibrahim Abu-Gheida, Akram Al Ibraheem, Kamal Al Rabii, Ala’a Farkouh, Mohammed Shahait

**Affiliations:** 1 Medical School, University of Jordan, Amman, Jordan; 2 Department of Medicine, Clemenceau Medical Center, Dubai, UAE; 3 Department of Medicine, Burjeel Medical City, Abu-Dhabi, UAE; 4 Department of Radiation Oncology, Cleveland Clinic Abu-Dhabi, Abu Dhabi, UAE; 5 Department of Nuclear Medicine, King Hussein Cancer Center, Amman, Jordan; 6 Department of Medicine, King Hussein Cancer Center, Amman, Jordan; 7 Department of Surgery, Clemenceau Medical Center, Dubai, UAE

## Abstract

**Background:**

Prostate cancer remains a major public health challenge in the Arab world with few population-based screening programmes, a high incidence of advanced disease at diagnosis, and limited patient access to sub-specialist care. A large number of patients diagnosed with prostate cancer use the (World Wide Web) internet to learn more about the disease and treatment options; however, material in the Arabic language is scarce. This study aims to objectively assess the quality and reliability of the information on YouTube™, which is the most globally used video platform, pertaining to prostate cancer videos published in Arabic.

**Methods:**

A total of 100 videos were identified by searching specific keywords in Arabic (Prostate cancer, prostate cancer treatment and prostate). Retrieved videos were analysed and categorised into four groups according to content as useful, misleading, personal experience, or irrelevant. Useful videos were assessed using the global quality scale (GQS) as a validated measure of quality, which is graded on a 5-point Likert scale, with 1 representing poor quality and 5 representing excellent quality. The modified DISCERN tool was used as a measure of reliability. The tool has a potential total score of 5 points, with higher scores indicating higher reliability.

**Results:**

Most of the speakers in these videos identified themselves as health workers (77%). Only 8% of the videos sources were hospital or medical organisations. Of the 100 retrieved videos, 86% were found to have useful content, while 14% were found to be misleading or irrelevant. The median GQS score of the useful videos was 4 (IQR: 4–5), while the median modified DISCERN tool was 4 (IQR: 3–4).

**Conclusion:**

To our knowledge, this is the first in-depth study to objectively assess the quality and reliability of information pertaining to prostate cancer in the Arabic language on YouTube™. More efforts are needed to improve the quality of prostate cancer educational materials and videos in the Arabic language on YouTube™. Patient focus groups are planned as the next step to address the information gap for patients with prostate cancer in the Arabic language.

## Introduction

Prostate cancer remains a major public health challenge in the Arab world with few population-based screening programmes, a high incidence of advanced disease at diagnosis, and limited patient access to sub-specialist care [1, 2]. It was estimated that approximately 80% of internet users have searched online about health topics, and 25% have watched an online video about health or medical topic [3]. The video-sharing platform, YouTube™, has become a source of information for patients seeking medical information, including prostate cancer [4]. However, material in the Arabic language is scarce.

The ability of the patient and caregiver to recognise the level of accuracy and reliability of YouTube™ videos are questionable, especially in the Arab World, which has one of the highest levels of illiteracy and low health literacy [5]. For example, 50% of people aged 50 years and above had inadequate health literacy [5].

High-quality and accurate videos and other visual materials might help patients to get a better understanding of their disease, and healthcare needs [6]. The quality of videos about prostate cancer and its management on YouTube™ has not been objectively assessed yet. This study aims to objectively evaluate the quality and reliability of the information on YouTube™ of prostate cancer videos published in Arabic.

## Materials and methods

### Search strategy

The terms in Arabic (Prostate cancer, prostate cancer treatment and prostate) were used to search YouTube™ (www.YouTube.com) (Supplementary Table 1). The default search setting on YouTube™ was used, which automatically sorts videos by relevance. Videos included were in Arabic, and others were excluded if they were recurrent in part or whole, not uploaded in Arabic language, or did not contain information on prostate cancer. A total of 100 videos were collected, over 4 months (26.06.2022–27.10.2022) during the searches for the search terms and were compiled into a playlist for evaluation.

Several video characteristics were abstracted, including the publisher, the speaker (health worker, non-medical), upload location, number of views, length (minutes), number of likes, number of comments, and days available on YouTube™.

### Quality analysis

A total of 100 videos were identified by searching specific keywords in Arabic (Prostate cancer, prostate cancer treatment and prostate). Retrieved videos were analysed and categorised into four groups according to content as useful, misleading, personal experience, or irrelevant. Useful videos were assessed using the global quality scale (GQS) as a validated measure of quality [7, 8], which is graded on a 5-point Likert scale, with 1 representing poor quality and 5 representing excellent quality. The modified DISCERN tool was used as a measure of reliability [7, 9, 10]. The tool has a potential total score of 5 points, with higher scores indicating higher reliability.

### Data analysis

Descriptive statistics were used to report the outcomes of this study. Continuous variables were reported as a mean, range and SD. Categorical variables were summarised as a number and percentages. The two-sample* t*-test was used to assess the quality analysis and misinformation tools, and a *p*-value of <0.05 was considered statistically significant. Mann-Whitney *U* test was also used to compare distributions when the data were not normally distributed.

## Results

A total of 110 videos were initially collected, and 10 videos in total were excluded. Seven were excluded due to different languages other than Arabic language, and three excluded for duplicated in part or whole. The majority of the included videos were published by independent users and news channels (85%), and most of the content created was from Egypt (24%). The median number of days videos were available on YouTube™ was 977.5 days (range, 1–4,160). The median length of the videos was 05:42 (00:12–25:52), and the median number of unique views was 6,547 (3–2,480,000). The median number of likes, dislikes, and comments was 60, 0 and 4, respectively (Table 1).

Most of the videos were presented by health professionals (77%), and most of the content was focusing on disease in general (general knowledge, screening, diagnosing and treatment briefly) (56%) (Table 2). The video content was labeled as useful in 84% of videos, misleading in 6%, irrelevant in 6%, and depicted personal experience in 4% of videos.

The median of the modified DISCERN score of videos presented by health workers was higher compared to videos presented by non-health workers (4 versus 2; *p* < 0.00001). A significant proportion of 78% of low-reliability videos (<=2) was presented by non-health workers. The GQS tool revealed that videos developed by health workers compared to videos presented by non-health workers had higher moderate to excellent levels of quality (80.5% versus 21.7%, *p* < 0.05) Table 3.

## Discussion

This is, as far as we are aware, the first YouTube cross-sectional study on prostate cancer that includes Arabic speakers. In this manuscript, the YouTube™ platform was used to collect videos pertaining to prostate cancer in Arabic language. We evaluated 100 videos with a cumulative duration of 11.2 hours and viewership of over 8 million and provided a detailed analysis of the YouTube™ videos as a source of medical information. Out of the 100 videos, 84% were useful, and 4% as personal experiences, indicating the platform is a great stage for broadcasting.

In the era of digital knowledge, online information is freely available and is influencing patient choices. There is a rapid growth in the number of videos relating to health issues posted on different digital platforms. This could reflect the strengths and weaknesses of such platforms as the most frequently accessed information may not be the best which could affect the patients’ outcomes including preventing or postponing seeking medical attention or accessing appropriate interventions in a timely manner. Lauckner and Whitten [11] confirmed that YouTube™ was more effective in enhancing comprehension and attitudes toward cancer risk reduction compared with other platforms, suggesting that while the message is important, the format by which it is delivered greatly matters.

Earlier studies examining YouTube™ videos pertaining to prostate cancer showed content relating to prostate-specific antigen (PSA) screening, and treatment options such as radiotherapy and surgery. Steinberg *et al* [12] reported on 51 reviewed YouTube™ videos that the information content regarding prostate cancer was fair or poor in 73% of the videos. While Loeb *et al* [13] used a validated framework for health care information to assess the quality of 150 prostate cancer information on YouTube™, and found that the median quality of the videos was rated as moderate and a significant number of videos about prostate cancer (77%) contained potentially misinformative and/or biased and poor content within the video or comments section. Interestingly, our study showed that videos about prostate cancer in Arabic were uploaded by medical personnel, and 92% were deemed useful. While videos were uploaded by non-medical professionals, 78% had low reliability, and only 56% were deemed useful. This shows the positive and critical role of specialists in the region to educate the public.

While Ayoub *et al* [14] showed that the videos about breast cancer in the Arabic language on YouTube™ were considered poorly informational and inaccurate on breast cancer in the Arab World. Our study shows that the videos about prostate cancer in the Arabic language were useful, especially when it was presented by health worker professionals.

The importance of systemically studying these videos stems from the fact that regular social media-related metrics such as the number of views and thumbs up on YouTube™ did not confirm the credibility of information. Surprisingly, the most viewed videos appeared to be those with the most questionable scientific merit. And the poor-quality videos had greater views than the videos rated to have good quality [6].

Factors affecting YouTube™ content accuracy are attributed to the high proportion of consumers uploading the video content. And more importantly, the most popular videos included commercials and advertisements, reflecting a biased opinion toward prostate cancer and PSA testing [5, 6]. Other factors affecting the accuracy of content are that YouTube™ is a globally used resource and videos may reflect standards for certain countries which may cause confusion among users seeking ‘accurate’ information, adding to the fact that YouTube™ does not have any strict regulations, and requires no formal identification to upload and publish videos [15]. For these reasons, anyone – experts, companies and laypeople – can publish content.

As we live in an era of abundant information, which can be accessible by any person despite socio-economic status and health literacy level. There is an exigent need to develop a special framework to ‘Fact Check’ these videos and grade the quality of the content based on the best practice guidelines. Also, it is crucial to adapt and validate new metrics for healthcare-related videos that would improve the patient's or lay person’s choice to choose videos with the most reliable content.

This study has several limitations including the fact that the uploaded video content and dates might not be aligned with the most updated recommendations for prostate cancer treatment and screening. Additionally, using a cross-sectional study is limited by the continuously changing content on the internet which cannot be accurately captured. Additionally, the median time the videos were on YouTube™ was 977.5 days, therefore, these may not be consistent with the most updated recommendations for PSA screening. Further studies could expand on the study sample size, enhance the search terminology, follow-up for more recently uploaded videos over time, and include a more detailed analysis of the comments.

## Conclusion

In the era where there is expanding use of digital media, information is freely accessible and multiple channels exist, videos remain one of the most powerful and effective platforms. Our study confirms the value and accuracy of YouTube™ videos addressing the screening and treatment of prostate cancer in Arabic language uploaded by healthcare workers. Nevertheless, there is a growing need to devise modalities to enhance the accuracy of content about prostate cancer messages posted on different digital platforms where patient groups actively engage, and where information is freely uploaded on multiple platforms. Public knowledge and skills need to be improved in a manner to identify credible information contained in YouTube™ as well as other digital platforms to help inform their decisions pertaining to prostate cancer.

The medical community needs to be engaged directly on social media platforms and provide a credible voice to the conversation and highlight reliable sources to the public from those sites providing misinformation, as physicians, and specialised allied healthcare professionals remain the single most credible voice in healthcare.

## Conflicts of interest and funding

The authors have no conflict of interest to disclose, and the work was not funded or supported by any organisation.

## Figures and Tables

**Table 1.** Parameters of 100 YouTube videos on prostate cancer in Arabic language.

Views, median (range)	6,547 (3–2,480,000)
Number of likes, median (range)	60 (0–80,0000)
Number of dislikes, median (range)	0
Number of comments, median (range)	4 (0–1,162)
Time, median (range)	05:42 (00:12–25:52)

**Table 2.** Characteristics of 100 YouTube videos on prostate cancer in Arabic language.

Type of provider, *n* (%)	
Medical advertisements	4 (4%)
Independent users	43 (43%)
Government/News outlet	42 (42%)
University	6 (6%)
Health information websites	5 (5%)
**Content of videos, *n* (%)**	
Prostate cancer in general	56 (56%)
Prostate cancer diagnosis/screening	24 (24%)
Prostate cancer treatment	20 (20%)

**Table 3.** Quality and misinformation analysis of 100 YouTube videos on prostate cancer in Arabic language.

	Medical speaker *N* = 77	Non-medical speaker *N* = 23
Modified discern, *n* (%)		
1	0 (0%)	8 (34.8%)
2	5 (6.4%)	10 (43.5%)
3	24 (31.2%)	4 (17.4%)
4	30 (39.0%)	0 (0%)
5	18 (23.4%)	1 (4.3%)
Median (IQR)	4	2
GQS, *n* (%)		
Poor	0 (0%)	2 (8.7%)
Generally poor	2 (2.6%)	7 (30.4%)
Moderate	13 (16.9%)	9 (39.1%)
Good	27 (35.1%)	4 (17.4%)
Excellent	35 (45.4%)	1 (4.3%)
Median (IQR)	4	3

**Supplementary Table 1.** Search strategy terms in Arabic.

English terminology	Arabic terminology
Prostate cancer	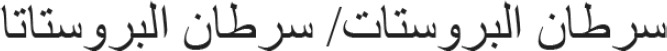
Prostate cancer treatment	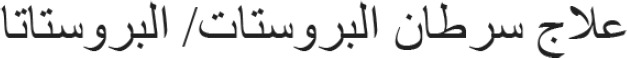
Prostate cancer diagnosis