# Quality and reliability of YouTube videos in Arabic as a source of patient information on prostate cancer

**DOI:** 10.3332/ecancer.2023.1646

**Published:** 2023-12-08

**Authors:** Laith Baqain, Deborah Mukherji, Humaid O Al-Shamsi, Ibrahim Abu-Gheida, Akram Al-Ibraheem, Kamal Al Rabii, Ala’a Farkouh, Mohammed Shahait

**Affiliations:** 1 Medical School, University of Jordan, Amman, Jordan; 2 Department of Medicine, Clemenceau Medical Center, Dubai, UAE; 3 Department of Medicine, Burjeel Medical City, Abu-Dhabi, UAE; 4 Department of Radiation Oncology, Cleveland Clinic Abu-Dhabi, Abu Dhabi, UAE; 5 Department of Nuclear Medicine, King Hussein Cancer Center, Amman, Jordan; 6 Department of Medicine, King Hussein Cancer Center, Amman, Jordan; 7 Department of Surgery, Clemenceau Medical Center, Dubai, UAE

**Keywords:** prostate cancer, YouTube, videos, Arabic, social media, misinformation

Akram Al Ibraheem is corrected to Akram Al-Ibraheem.

